# Differential roles of confidence and competence in the association between understanding and enjoyment of physical activity: developmental and demographic moderators in children and adolescents

**DOI:** 10.3389/fpubh.2026.1875229

**Published:** 2026-09-30

**Authors:** Tim Vernon, Maxine Gregory, Stuart Bonner, Simon Goldsmith, Orla Turnbull

**Affiliations:** 1 School of Sport and Physical Activity, Sheffield Hallam University, Sheffield, United Kingdom; 2 Mosodi Research, Manchester, United Kingdom

**Keywords:** children and young people, confidence, enjoyment, mediation, physical activity, physical literacy, self-determination theory

## Abstract

**Introduction:**

Enjoyment is widely recognized as a key determinant of young people's engagement in physical activity (PA) and their longer-term active behaviors. However, less is known about the cognitive and affective mechanisms that underpin enjoyment, or how these vary across groups of pupils. This study examined the association between pupils' understanding of the benefits of PA, their confidence and perceived competence, and their enjoyment of PA, and explored whether these patterns differed according to gender, ethnicity, physical activity level, SEN status, or school setting.

**Methods:**

A cross-sectional survey was completed by 637 pupils aged 8–16 years from five schools across England. Enjoyment, confidence, competence, and understanding of PA were each assessed using a single-item measure, administered alongside the Physical Activity Questionnaire for Children (PAQ-C). A path analysis model tested whether confidence and competence mediated the association between understanding and enjoyment, and multi-group structural equation modeling examined moderation; subgroup analyses were treated as exploratory.

**Results:**

Understanding was indirectly associated with enjoyment through both confidence and competence, with confidence the stronger mediator across the full sample and for most subgroups; both indirect effects were statistically significant. Moderation analyses indicated that these patterns varied across physical activity level, school setting, and ethnicity, but not gender or SEN status. The relative contribution of confidence and competence shifted across school settings: confidence was the dominant mediator in secondary and special school settings, whereas in primary settings the two pathways did not differ reliably. Active pupils showed stronger confidence-mediated associations than less active peers. Apparent differences across ethnic groups were largely attributable to school composition.

**Discussion:**

The study contributes to self-determination theory and physical literacy perspectives by showing how cognitive understanding is associated with the affective mechanisms linked to enjoyment, and how these associations vary across school settings. Practically, the findings suggest that PA provision may benefit from prioritizing confidence-building in secondary settings, competence-building in primary settings, and inclusive, culturally responsive pedagogies throughout; these implications follow from cross-sectional associations rather than demonstrated intervention effects. By centring enjoyment and its psychological antecedents, schools can support positive, sustainable relationships with physical activity that extend beyond the classroom.

## 1 Introduction

Engagement in physical activity (PA) supports not only immediate involvement in exercise and sport but also the development of long-term activity habits and broader physical, social, and emotional outcomes (1). Enjoyment sits at the center of this process: early observational frameworks, such as Leuven's Scale of Active Engagement (2), emphasize emotional involvement, focus, and persistence, qualities that are closely tied to whether children experience movement as pleasurable and are equally relevant in PA contexts where children feel free to explore movement, take risks, and derive satisfaction from their efforts (3, 4).

Within this broader landscape, enjoyment has emerged as a central construct in understanding how young people engage with PA and sustain active behaviors over time, with a substantial body of research demonstrating that when children enjoy being active, they are more likely to participate regularly in PA, both within and beyond school settings (5, 6). This relationship is particularly salient given global concerns about declining PA levels among children and adolescents, with recent estimates indicating that more than 80% of young people fail to meet recommended daily activity guidelines (7).

Despite this, many school-based PA initiatives prioritize quantitative indicators, frequency, intensity, and duration, over the affective experiences associated with participation. Programmes such as The Daily Mile, for example, focus on ensuring children complete 15 min of running or jogging each day, with the primary aim of increasing overall PA levels (8). Similarly, public health guidelines emphasize achieving 60 min of moderate-to-vigorous physical activity (MVPA) per day (9), prioritizing dose-response relationships linked to physical health outcomes. While these approaches are valuable, they risk overlooking the emotional, motivational, and psychological dimensions of PA that are critical for sustained engagement (10). Evidence suggests that enjoyment, rather than MVPA volume alone, predicts both short-term participation and longer-term behavioral outcomes (6), functioning as a gateway to building motivation for maintaining healthy PA levels into adolescence and adulthood. Keeping enjoyment in view as the primary outcome of interest, the remainder of this introduction considers the psychological resources that shape it.

The theoretical basis for the role of enjoyment is well established in Self-Determination Theory (SDT), which posits that intrinsic motivation is driven by the satisfaction of three basic psychological needs: autonomy, competence, and relatedness (11). Autonomy refers to a sense of volition and ownership over one's participation; competence to feeling capable and effective in the activity; and relatedness to experiencing meaningful social connection with others. These needs are met when young people feel a sense of control over their participation, perceive themselves as capable, and experience positive social connections. When these conditions are present, children report higher enjoyment and are more likely to engage positively with PA across settings (12, 13). The present study focuses primarily on the competence strand of this framework, and on the closely related construct of confidence, because these constructs are most directly implicated in how understanding translates into enjoyment; however, autonomy and relatedness remain relevant throughout, since the social comparison, choice, and belonging that characterize PA settings shape the climate within which competence and confidence develop. Consistent with this, Adank et al. (14) found that enjoyment among primary-aged children was enhanced when adults offered opportunities for cooperation (relatedness), choice (autonomy), and skill development at their own pace (competence), illustrating how all three needs operate together to support enjoyment. Enjoyable early experiences are also linked to more positive self-concept and to continued engagement in sport and PA as children age (15), suggesting enduring impacts on health and wellbeing trajectories.

The needs identified by SDT find clear parallels in the construct of physical literacy, which extends this motivational framework by situating competence and confidence within a broader commitment to understanding and valuing physical activity throughout life (16–18). Within this framework, understanding refers to children's awareness of why exercise and sport are beneficial and how that awareness shapes their orientation toward PA. Although often overlooked in empirical models of PA engagement, this cognitive component plays a foundational role in how young people interpret their experiences, make sense of expectations, and navigate opportunities for participation. Understanding is conceptually distinct from confidence and competence, reflecting a cognitive appraisal of the value and purpose of physical activity rather than a self-evaluation of capability or skill (17, 19). By nurturing pupils' understanding of why PA matters, practitioners can build the cognitive foundations that support confidence, competence, and ultimately enjoyment (20).

To avoid conceptual ambiguity, and because the analyses that follow depend on clear separation of these constructs, the four central constructs are defined as follows. *Understanding* denotes a pupil's cognitive awareness of the value and benefits of physical activity (i.e., knowing why exercise and sport are good for them). *Confidence* denotes a pupil's belief in their own ability to take part in physical activity, a self-efficacy-like appraisal of “can I do this?” *Competence* denotes a pupil's perception of their skill, capability, or ease in physical activity, an appraisal of “how good or able am I?” *Enjoyment* denotes the positive affect or pleasure a pupil experiences during physical activity. Confidence and competence are related but distinct: a pupil may feel broadly confident about taking part without rating themselves as highly skilled, and the central question of this study is whether these two appraisals play different roles in linking understanding to enjoyment.

Despite widespread agreement on the importance of enjoyment as a predictor of sustained engagement in PA, there remains limited understanding of the specific factors that drive enjoyment across different groups of young people. Enjoyment is often treated as a unidimensional outcome, yet it is a complex, multifaceted experience shaped by autonomy, perceived competence, social dynamics, and the relevance or inclusivity of the activity (21, 22). Existing studies frequently report overall levels of enjoyment without sufficiently disaggregating findings by demographic factors such as gender, ethnicity, or disability status, masking important nuances in how different children experience PA.

Emerging evidence suggests that enjoyment in PA is not equally distributed, with girls often reporting lower enjoyment and engagement than boys, particularly during adolescence (15) and differences linked to activity types, social climate, and definitions of success. Children from ethnically minoritised backgrounds may face cultural or structural barriers that shape their sense of inclusion or enjoyment (23), while disabled pupils often encounter low expectations, poorly adapted opportunities, or limited access to meaningful participation (24). Although frameworks such as physical literacy and SDT offer valuable insights, there remains a need for more fine-grained, intersectional research that examines how different pupils interpret and value their PA experiences. Without such understanding, interventions risk reinforcing dominant norms and practices that marginalize certain groups. Moreover, while enjoyment is widely recognized as a key driver of engagement, far less attention has been given to the cognitive perceptions that underpin it, specifically, pupils' understanding of why physical activity is beneficial and how this shapes the motivational processes that ultimately influence enjoyment.

The present study sought to develop and test a model of enjoyment in PA using large-scale student survey data. Building on theoretical perspectives from self-determination theory and physical literacy, and responding to calls for more nuanced, intersectional analyses of PA experiences, the study aimed to identify the cognitive and affective factors associated with pupils' enjoyment and to examine how these patterns vary across demographic and contextual groups. Specifically, the study addressed three research questions: (i) To what extent is pupils' understanding of physical activity associated with enjoyment through the mediating roles of confidence and competence? (ii) Do these mediation patterns differ according to gender, ethnicity, physical activity level, or school setting? (iii) How do patterns of enjoyment and its psychological antecedents vary across pupil subgroups? Based on existing theory and empirical evidence, it was hypothesized that: (a) confidence and competence would significantly mediate the association between understanding and enjoyment; and (b) confidence would emerge as the stronger mediator across the full sample. Because directional, group-specific predictions are not well supported by existing evidence, the subgroup questions (ii and iii) were treated as exploratory rather than confirmatory: we did not predict in advance which paths would differ across which groups, and instead examined whether the mediation patterns varied across demographic and contextual groups and, where they did, characterized the nature of that variation. Together, these questions aim to advance understanding of the psychological processes that underpin enjoyment in PA and to inform the development of more inclusive, engaging, and equitable physical activity provision for young people.

## 2 Materials and methods

### 2.1 Design

The study employed a cross-sectional survey design to examine the cognitive and affective correlates of pupils' enjoyment of physical activity (PA), and to explore whether these relationships varied across demographic and contextual groups. This design enabled the simultaneous estimation of direct, indirect, and moderated associations, while precluding inferences about causal direction. Analyses are best characterized as path analysis within a structural equation modeling (SEM) framework, given that all constructs were assessed using single-item indicators. The implications of this analytic choice for model fit evaluation are addressed in sections 2.3 and 3.5.

### 2.2 Participants

A total of 637 pupils took part in the study, drawn from five schools across England. Participants ranged in age from 8 to 16 years and represented a broad cross-section of demographic backgrounds. The full sample comprised 310 boys (48.7%), 294 girls (46.2%), and 33 pupils (5.2%) who identified as “Other” (*n* = 14) or “Prefer not to say” (*n* = 19).

Ethnicity was self-reported using six categories aligned with the UK Office for National Statistics; group sizes are reported in Table 1. For multi-group SEM, only the three largest groups were retained to ensure adequate subgroup sizes: White British or English (*n* = 221), Asian or British Asian (*n* = 178), and Black or Black British (*n* = 105).

**Table 1:** Group-specific descriptive statistics.

A. Gender	*n*	PAQ-C *M* (SD)	Enjoyment *M* (SD)	Confidence *M* (SD)	Competence *M* (SD)	Understanding *M* (SD)
Boys	310	3.15 (0.86)	4.16 (1.03)	3.95 (0.99)	3.67 (1.06)	4.39 (0.76)
Girls	294	2.73 (0.86)	3.59 (1.13)	3.37 (1.16)	3.41 (1.07)	4.21 (0.92)
Other	14	2.95 (1.21)	2.71 (1.49)	2.79 (1.53)	2.71 (1.33)	3.64 (1.28)
Prefer not to say	19	3.08 (1.00)	3.26 (1.37)	3.32 (1.20)	3.21 (1.32)	3.84 (1.21)
B. PAQ-C activity level						
Active	328	3.61 (0.54)	4.39 (0.89)	4.15 (0.92)	3.93 (1.01)	4.52 (0.75)
Less active	276	2.15 (0.44)	3.29 (1.07)	3.11 (1.05)	3.08 (0.97)	4.04 (0.88)
C. School setting						
Primary	149	3.61 (0.77)	4.23 (1.04)	4.05 (1.04)	3.74 (1.16)	4.50 (0.92)
Secondary or special	488	2.75 (0.83)	3.72 (1.16)	3.52 (1.13)	3.45 (1.07)	4.20 (0.85)
D. SEN status						
No SEN	509	3.00 (0.88)	3.91 (1.11)	3.70 (1.10)	3.56 (1.08)	4.32 (0.84)
SEN	128	2.75 (0.92)	3.56 (1.28)	3.40 (1.21)	3.33 (1.16)	4.07 (0.99)
E. Ethnicity						
Asian or British Asian	178	3.13 (0.91)	4.11 (0.99)	3.83 (1.06)	3.62 (1.05)	4.45 (0.69)
Black or Black British	105	2.79 (0.83)	3.72 (1.12)	3.62 (1.01)	3.51 (1.04)	4.27 (0.86)
Mixed race	62	3.05 (0.80)	3.95 (1.27)	3.71 (1.26)	3.63 (1.13)	4.21 (1.06)
None of these	40	3.15 (0.73)	3.92 (1.19)	3.80 (1.14)	3.98 (1.03)	4.35 (0.80)
White (British or English)	221	2.83 (0.95)	3.63 (1.23)	3.44 (1.22)	3.33 (1.16)	4.11 (0.98)
White (not British or English)	31	2.83 (0.66)	3.87 (0.88)	3.74 (0.86)	3.42 (0.85)	4.48 (0.57)

Other gender categories and smaller ethnicity groups were retained in descriptive analyses but excluded from multi-group SEM. PAQ-C active/less active classification used gender-specific thresholds (boys ≥ 2.9, girls ≥ 2.7). n for PAQ-C groups sums to 604 as 33 pupils identifying as Other or Prefer not to say gender were excluded from the activity classification. Secondary or Special combines secondary school (*n* = 406) and special school (*n* = 82) pupils. PAQ-C M (SD) is reported for all groups; for the PAQ-C Activity Level panel, scores reflect the continuous PAQ-C variable within each dichotomised group.

The sample was drawn from two primary schools, two secondary schools, and one special secondary school. To preserve the anonymity of participating institutions, schools are identified throughout by letter: Schools A and B (primary), Schools C and D (secondary), and School E (special). Pupil numbers varied considerably across the five settings: the primary schools contributed 98 (School A) and 51 (School B) pupils respectively (*n* = 149), the mainstream secondary schools contributed 336 (School C) and 70 (School D) pupils (*n* = 406), and the special school contributed 82 pupils (School E). School C therefore accounted for 52.7% of the full sample and 68.9% of the combined Secondary or Special group.

Primary pupils were drawn from Years 5 to 6, and secondary and special school pupils from Years 7 to 11. Participant characteristics by school, including composition by gender, SEN status, ethnicity and activity level, are presented in Table 2.

**Table 2:** Participant characteristics by school.

School	Setting	n	Year groups	Boys	Girls	Other/PNS	SEN	Asian/British Asian	Black/black British	Mixed Race	None of these	White (British/English)	White (other)	% Active
A	Primary	98	5–6	35	52	11	6	63	4	11	9	7	4	85.1
B	Primary	51	5–6	27	22	2	6	0	1	2	1	47	0	85.7
C	Secondary	336	7–11	155	170	11	40	60	98	41	22	91	24	46.8
D	Secondary	70	7–11	31	37	2	5	53	1	6	6	2	2	44.1
E	Special	82	7–11	62	13	7	71	2	1	2	2	74	1	40.0
**Total**	—	**637**	—	**310**	**294**	**33**	**128**	**178**	**105**	**62**	**40**	**221**	**31**	**54.3**

Schools are identified by letter to preserve institutional anonymity. Values are counts of pupils unless otherwise indicated. Other / PNS combines pupils identifying as “Other” (*n* = 14) and those preferring not to state their gender (*n* = 19); gender categories sum to each school's n. SEN status was self-reported (section 2.2). % Active is the proportion classified as Active using gender-specific PAQ-C thresholds, calculated among pupils who could be classified (section 2.4). Schools differ markedly in composition: schools B and E are predominantly White British, Schools A and D predominantly Asian or British Asian, and School E has substantially fewer girls than the other settings. The two primary schools (A, B) have much higher proportions of Active pupils than the secondary and special settings. These compositional differences are relevant to the interpretation of the moderation analyses and are discussed in sections 3.11, 3.12 and 4. Values in the Total row are presented in bold to distinguish aggregate sample-wide statistics from the individual school-level counts above them.

School E was a co-educational community special school catering for pupils aged 11–19 who have a moderate learning difficulty and/or an additional need such as an autism spectrum condition, with pupils educated in either mixed groups or a dedicated autism spectrum condition provision.

Special educational needs status was self-reported: pupils indicated whether they considered themselves to have a special educational need or disability. Of the 128 pupils who identified as having SEN, 71 (55.5%) attended the special school and 57 (44.5%) were drawn from the mainstream primary and secondary settings, representing 10.3% of mainstream pupils. Of the 82 special school pupils, 11 (13.4%) did not self-identify as having SEN, despite the school's admission arrangements, under which pupils are placed following an Education, Health and Care Plan. The SEN variable analyzed here therefore indexes pupils' self-identification rather than administrative designation; the validity implications of this are examined in the Limitations, and a sensitivity analysis recoding special school pupils by placement status is reported in section 3.12. The SEN group is also heterogeneous, combining pupils in a specialist setting with pupils identifying as having SEN within mainstream provision. School setting was initially coded as Primary (*n* = 149), Secondary (*n* = 406), or Special (*n* = 82). For multi-group SEM analyses, Secondary and Special school pupils were combined into a single group (Secondary or Special; *n* = 488) to ensure adequate subgroup sizes for reliable model estimation.

### 2.3 Measures

Four psychological constructs central to pupils' experiences of physical activity, Enjoyment, Confidence, Competence, and Understanding, were each assessed using a single-item measure aligned with those used in the Sport England Child Question Bank (25). Pupils responded to statements reflecting their broader perceptions of exercise and sport using a five-point Likert scale ranging from 1 = *Strongly disagree* to 5 = *Strongly agree*. Items were designed to be clear, age-appropriate, and easily interpretable for pupils aged 8–16. The exact item wording, response scale, source, and intended interpretation of each construct are summarized in Table 3. Higher scores indicated more positive perceptions on each construct.

**Table 3:** Measurement of psychological constructs (single-item indicators).

Construct	Item wording	Response scale	Source	Interpretation	Key limitation
Enjoyment	“I enjoy taking part in exercise and sports”	1 = Strongly disagree to 5 = Strongly agree	Sport England child question bank (24)	Positive affect/pleasure experienced during PA; higher = more enjoyment	Single item; global affective judgement only
Confidence	“I feel confident when I exercise and play sports”	1 = Strongly disagree to 5 = Strongly agree	Sport England child question bank (24)	Belief in one's ability to take part in PA; higher = more confidence	Single item; not separated from related self-efficacy constructs
Competence	“I find exercise and sports easy”	1 = Strongly disagree to 5 = Strongly agree	Sport England child question bank (24)	Perceived skill/capability/ease in PA; higher = greater perceived competence	May partly index perceived task difficulty rather than skill
Understanding	“I understand why exercise and sports are good for me”	1 = Strongly disagree to 5 = Strongly agree	Sport England child question bank (24)	Cognitive awareness of the value/benefits of PA; higher = greater understanding	Ceiling-leaning distribution (*M* = 4.27, SD = 0.88); limited upper-end variance

All items refer to exercise and sport in general and were not restricted to physical education lessons. PA, physical activity.

Single-item indicators were selected for several reasons. The constructs assessed are concrete, unidimensional, and within the comprehension of the target age range, characteristics identified as preconditions for valid single-item measurement (26, 27), and the items align with Sport England's validated Child Question Bank (25). The case for single-item measurement of narrow constructs has also strengthened in recent years (28), with youth PA studies showing acceptable convergent validity against multi-item scales (26, 27, 29); of particular relevance, Martela and Ryan (30) validated single-item scales for the three self-determination theory needs, including competence, showing performance comparable to established multi-item measures. Finally, in school-based research with pupils aged 8–16, survey brevity reduces response fatigue and item non-response.

Two measurement caveats specific to the items used here are relevant to interpretation. First, the competence item (“I find exercise and sports easy”) indexes perceived ease, which overlaps with but is not identical to perceived skill or capability: a pupil may find exercise and sport easy because they are capable, but also because they find such activity undemanding or comfortable, and these appraisals need not coincide. Because the item refers to exercise and sport in general rather than to any specific activity, it is unlikely to reflect the perceived difficulty of a particular task; the relevant consideration is therefore the more general distinction between finding activity easy and judging oneself skilled at it. This should be borne in mind when interpreting the competence pathway, and is revisited in the Limitations. Second, the understanding item showed a relatively high mean and a ceiling-leaning distribution (*M* = 4.27, SD = 0.88; skewness up to −1.60; see section 3.1), indicating limited variability at the upper end of the scale. While the item retained sufficient spread to support the mediation model, the restricted variance may attenuate observed associations involving understanding, and replication with a more graduated or multi-item measure of understanding is desirable.

A consequence of single-item measurement is that measurement error cannot be modeled separately from the structural paths, so the analyses are best characterized as path analysis within an SEM framework rather than full latent-variable SEM; standard global fit indices (CFI, RMSEA, SRMR) are correspondingly uninformative about measurement quality and are reported for transparency only (section 3.5).

Pupils responded about exercise and sport in general rather than about physical education (PE) specifically, and the survey was not administered within PE lessons; the items were designed to capture pupils' broader orientations toward exercise and sport. The findings therefore carry relevance for PE practitioners given the school's central role in shaping young people's physical activity attitudes and behaviors, but claims about PE practice are framed cautiously throughout, since the data speak most directly to pupils' general orientations toward physical activity and sport rather than to lesson-specific experience.

### 2.4 Physical activity level (PAQ-C)

Physical activity level was assessed using the Physical Activity Questionnaire for Children (PAQ-C), a widely used and validated self-report instrument designed to capture general levels of moderate-to-vigorous physical activity over the previous seven days (31). The PAQ-C provides a composite score from 1 to 5, with higher scores indicating greater habitual physical activity, and has demonstrated good reliability and validity in children and adolescents.

The PAQ-C was retained across the full 8–16 age range, rather than switching to the Physical Activity Questionnaire for Adolescents (PAQ-A) for older pupils, to ensure comparability across year groups within the same analytic model; the two instruments share the majority of items, and the PAQ-C has been normed in samples extending into adolescence (32).

Because the PAQ-C references the previous seven days, scores are sensitive to the timing of administration. Data were collected during the autumn term (September–December) of 2025. Because all pupils were surveyed within the same term, differences in timing across pupils were minimal, and seasonal variation is therefore unlikely to account for the between-group differences or mediation patterns that form the substance of the analyses. Consistent with previous research (32), scores were dichotomised using gender-specific thresholds to categorize pupils as Active or Less Active. Boys with PAQ-C scores of ≥2.9 and girls with scores of ≥2.7 were classified as Active (*n* = 328), with those below the threshold categorized as Less Active (*n* = 276). 33 pupils who identified as Other or Prefer not to say gender could not be classified using these gender-specific thresholds and were therefore excluded from PAQ-C activity-level analyses.

### 2.5 Procedure

Written gatekeeper consent was provided by each participating school. Information about the study was distributed to children and parents/carers through a series of age-appropriate information sheets, and an (opt-out) parental consent procedure was used, whereby parents/carers were given the opportunity to withdraw their child from the study. Pupils themselves provided assent prior to completing the survey and were informed that they could decline to take part, omit any question, or withdraw at any point during data collection without consequence. An opt-out option was provided for every demographic question should respondents prefer not to disclose this information. Institutional ethics approval was granted for the study on 4th March 2025 (Converis Number ER74231596).

An online survey was distributed to the five participating schools, who were supported in determining the most appropriate method of administration given their individual circumstances. All responses were anonymous, and no identifiable information was collected, with completed surveys stored securely in accordance with institutional data protection procedures.

### 2.6 Data preparation and analytic strategy

Data were screened for missing values, normality, and outliers prior to analysis. Missingness was minimal and handled using listwise deletion. All variables demonstrated acceptable skewness and kurtosis for maximum likelihood estimation (|skewness| < 2, |kurtosis| < 7; (33)).

Analyses were conducted in R 4.5.2 (34) using the lavaan package (35). Descriptive statistics and bivariate correlations were first computed to provide an overview of the data and to examine initial associations among the four psychological constructs and PAQ-C scores.

A parallel mediation model was estimated to test whether Confidence and Competence mediated the association between Understanding and Enjoyment. Standardized coefficients (β) were used for interpretation. Indirect effects were evaluated using non-parametric bootstrapping with 5,000 resamples to generate percentile 95% confidence intervals. To examine shared variance between the mediators, an alternative specification freeing the residual covariance between Confidence and Competence was also estimated. The relative strength of the two indirect pathways was formally compared using a bootstrap contrast.

Moderated mediation was examined using multi-group SEM. Separate models were estimated for gender, ethnicity, PAQ-C activity level, school setting, and SEN status. Moderation was assessed using likelihood ratio tests comparing constrained models (all structural paths held equal across groups) with unconstrained models (paths free to vary). Where overall moderation was detected, univariate score tests were used to identify the specific paths contributing to group differences. These subgroup analyses were exploratory.

Pupils were nested within five schools of highly uneven size, and all focal constructs were measured at the individual level, with no school-level predictors in the model. Multilevel modeling was not appropriate with so few clusters (36, 37); intraclass correlations and design effects were therefore computed, and the mediation model was re-estimated with cluster-robust standard errors as a robustness check. School setting was additionally incorporated as a grouping variable in multi-group SEM to examine contextual variation.

Because the school-setting model combined mainstream secondary and special school pupils, two sensitivity analyses were conducted: one comparing Primary with mainstream Secondary pupils only, and one estimating a three-group model (Primary, Secondary, Special). A further sensitivity analysis re-estimated the ethnicity model within a single school.

Effect sizes for group comparisons are reported as Cohen's d with statistical significance set at α =.05 for all analyses. Global model fit indices are reported for transparency only.

## 3 Results

### 3.1 Descriptive statistics

Descriptive statistics and correlations for all study variables are presented in Table 4. Across the full sample (*N* = 637), pupils reported moderately high levels of all four constructs and moderate habitual activity (PAQ-C M = 2.95, SD = 0.89). All variables showed acceptable skewness and kurtosis for maximum likelihood estimation (33). The psychological constructs were positively and significantly intercorrelated (all *p* < 0.001), most strongly between Enjoyment and Confidence (*r* = 0.72) and least between Understanding and Competence (*r* = 0.43).

**Table 4:** Overall descriptive statistics and correlations for study variables (*N* = 637).

Variable	M	SD	1	2	3	4	5
1. Enjoyment	3.84	1.15	—	0.72	.63	0.56	0.53
2. Confidence	3.64	1.13		—	0.66	0.46	0.53
3. Competence	3.52	1.10			—	0.43	0.45
4. Understanding	4.27	0.88				—	0.33
5. PAQ-C	2.95	0.89					—

All correlations significant at *p* < 0.001. PAQ-C, Physical Activity Questionnaire for Children; higher scores indicate greater habitual physical activity.

Group-specific descriptive statistics are presented in Table 1. Boys reported significantly higher Enjoyment, Confidence, Competence, and Understanding than girls (all *p* ≤ 0.011, ds = 0.21–0.54), as well as higher PAQ-C scores (*d* = 0.48), consistent with established gender differences in habitual physical activity among children and adolescents (38); some of the gender differences in the psychological constructs may therefore partly reflect differences in activity level rather than gender itself. Pupils identifying as “Other” (*n* = 14) or “Prefer not to say” (*n* = 19), retained in Table 1 for transparency, reported notably lower scores across all constructs, with the “Other” group scoring a full point below the male mean on Enjoyment (2.71 vs. 4.16). Notably, this was not accompanied by lower habitual activity: mean PAQ-C scores in these groups (2.95 and 3.08 respectively) were comparable to the full-sample mean of 2.95 and higher than that of girls (2.73), so their lower enjoyment, confidence, and competence cannot be attributed to doing less physical activity. Small sample sizes preclude formal comparison, and the data cannot establish whether this reflects genuine differences, social desirability, or response style; the pattern nonetheless highlights an equity concern warranting adequately powered research.

The largest group differences were by activity level: active pupils scored higher than Less Active pupils on all four variables (all *p* < 0.001), with large effects for Enjoyment, Confidence, and Competence (ds = 0.86–1.12) and a moderate effect for Understanding (*d* = 0.58). Because the groups also differ substantially in mean PAQ-C by construction, and the design is cross-sectional, these differences may partly reflect consequences rather than causes of engagement. Primary pupils scored higher than Secondary or Special pupils across all variables (all *p* ≤ 0.007; medium effects for Enjoyment and Confidence), but also reported PAQ-C scores approximately one standard deviation higher (*d* = 1.06), so the higher construct scores in primary settings partly track this group's higher activity levels; this school-setting/activity confound is examined in sections 4.3 and 4.4. Pupils with SEN scored significantly but modestly lower than those without on all four variables (all *p* ≤ 0.038, ds = 0.21–0.29), alongside slightly lower PAQ-C scores (*d* = 0.28).

Pairwise effect sizes for ethnicity are presented in Table 5. One-way ANOVAs indicated significant overall differences across the three analytic groups on all four variables (all *p* ≤ 0.027), driven primarily by Asian or British Asian pupils scoring higher than White British or English pupils (all four constructs, *p* ≤ 0.022); Black or Black British and White pupils did not differ significantly on any variable (all *p* > 0.33). Notably, Asian pupils reported higher activity than both Black (*d* = 0.38) and White pupils (*d* = 0.31) whereas Black and White pupils were near-identical (*d* = −0.05), so the ethnicity differences in the psychological constructs are not straightforwardly explained by activity level.

**Table 5:** Pairwise Cohen's *d* effect sizes for ethnicity groups (reference group = white British/English).

Ethnicity group	*N*	Enjoyment	Confidence	Competence	Understanding
Asian/British Asian	178	**0.42**	**0.33**	**0.27**	**0.38**
Black/Black British	105	0.08	0.15	0.16	0.17
Mixed race	62	0.27	0.20	0.26	0.10
None of these	40	0.25	0.22	**0.56**	0.17
White (not British/English)	31	0.11	0.25	0.08	0.40

Effect sizes in bold indicate d ≥ 0.30. All effect sizes calculated using White (British or English) as the reference group.

### 3.2 Associations between psychological constructs and physical activity level

Bivariate correlations indicated that PAQ-C scores were significantly associated with all four psychological constructs (all *p* < 0.001), with the strongest associations observed for Enjoyment (*r* = 0.53) and Confidence (*r* = 0.53), followed by Competence (*r* = 0.45) and Understanding (*r* = 0.33). Enjoyment alone accounted for 28.4% of the variance in PAQ-C scores (β = 0.533, F(1,635) = 251.4, *p* < 0.001), suggesting a robust association between pupils' enjoyment of physical activity and their habitual activity levels.

A multiple regression examined the unique contributions of all four constructs to PAQ-C scores. The overall model was significant, F(4,632) = 79.23, *p* < 0.001, explaining 33.4% of the variance. Enjoyment (β = 0.271, *p* < 0.001) and Confidence (β = 0.269, *p* < 0.001) emerged as equally strong independent predictors, with Competence making a smaller but significant contribution (β = 0.093, *p* = 0.041). Understanding was not a significant independent predictor of PAQ-C once the affective variables were accounted for (β = 0.015, *p* = 0.704). These findings suggest that enjoyment and confidence are the most proximal psychological correlates of habitual physical activity, while understanding may operate more distally, associated with activity levels indirectly through its relationship with confidence and competence rather than predicting activity behavior independently.

### 3.3 Regression model

A multiple regression was conducted to examine whether Confidence, Competence, and Understanding were associated with Enjoyment. The overall model was significant, F(3,633) = 335.10, *p* < 0.001, explaining 61.4% of the variance in Enjoyment. All three predictors made significant unique contributions: Confidence (β = 0.47, *p* < 0.001), Understanding (β = 0.25, *p* < 0.001), and Competence (β = 0.22, *p* < 0.001). Confidence was the strongest predictor, with Understanding and Competence contributing comparably. These results supported the specification of a parallel path analysis model with Confidence and Competence as mediators of the Understanding-Enjoyment relationship.

### 3.4 Parallel mediation model

A parallel path analysis model was estimated to examine whether Confidence and Competence mediated the association between Understanding and Enjoyment. Standardized and unstandardised path coefficients, indirect effects, and bootstrapped 95% confidence intervals (5,000 samples) are presented in Table 6, and the standardized solution is displayed in Figure 1.

**Figure 1:**
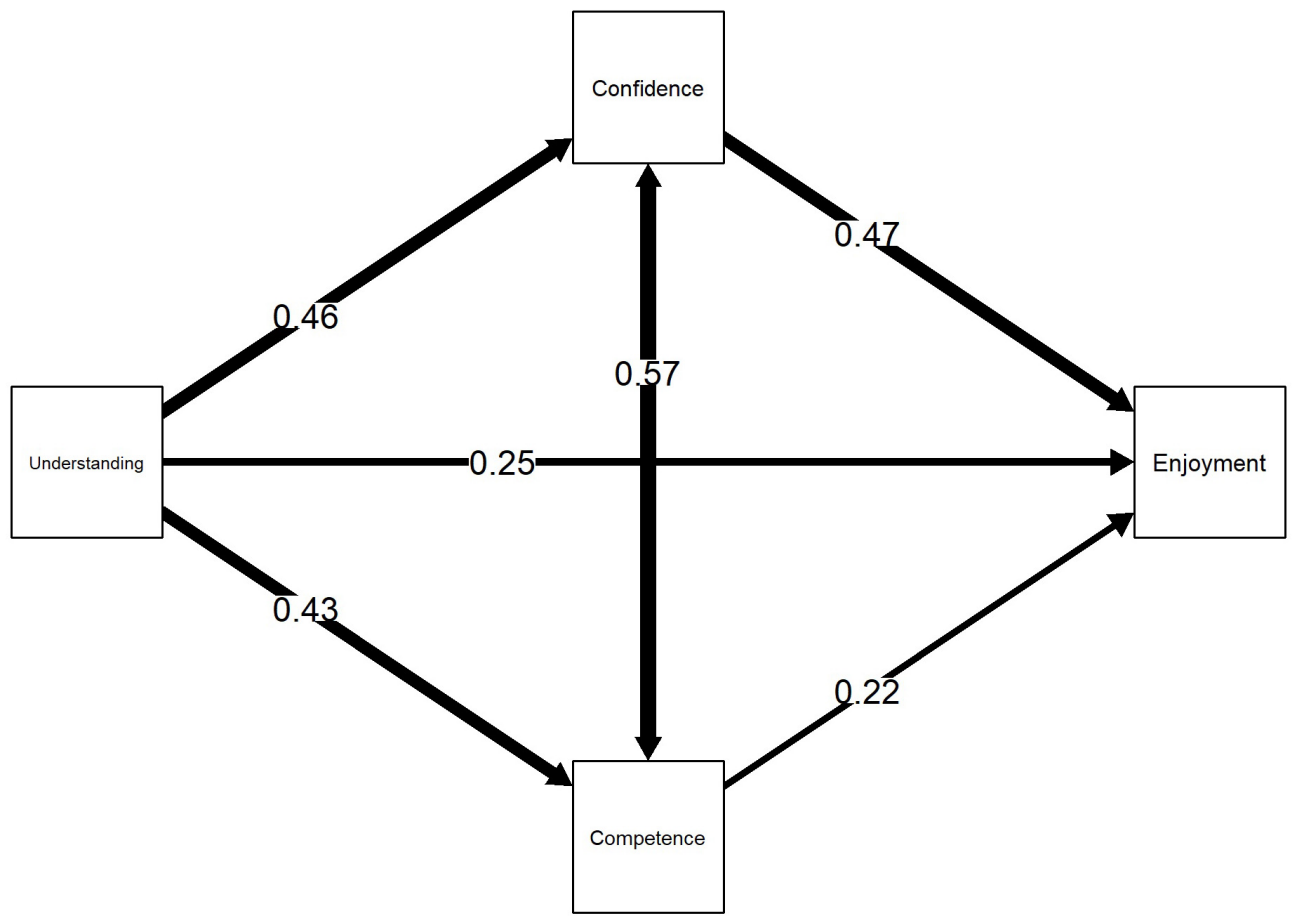
Standardized path coefficients for the parallel mediation model of understanding, confidence, competence, and enjoyment (*N* = 637). Values are standardized coefficients from the model in which the residual covariance between Confidence and Competence is freely estimated (section 3.5). The double-headed arrow between Confidence and Competence denotes their estimated residual correlation (0.57), not a directional path. Arrow thickness is proportional to coefficient magnitude. All coefficients *p* < 0.001. Figure generated from lavaan model output.

**Table 6:** Standardized path coefficients and indirect effects for the understanding mediation model.

Path	Std. β	Unstd. estimate	95% CI (Unstd.)
Understanding → confidence (a1)	0.461	0.596	[0.486, 0.699]
Understanding → competence (a2)	0.426	0.533	[0.442, 0.613]
Confidence → enjoyment (b1)	0.467	0.474	[0.381, 0.567]
Competence → enjoyment (b2)	0.217	0.228	[0.140, 0.316]
Understanding → enjoyment (direct, c′)	0.248	0.326	[0.240, 0.411]
Indirect via confidence	0.216	0.283	[0.206, 0.369]
Indirect via competence	0.093	0.122	[0.073, 0.173]
Contrast (confidence – competence indirect)	0.123	0.161	[0.053, 0.272]
Total Indirect	0.308	0.404	[0.331, 0.481]
Total Effect	0.557	0.731	[0.649, 0.806]

Standardized coefficients are from the model in which the residual covariance between Confidence and Competence is freely estimated (section 3.5). Unstandardised estimates and 95% CIs are from non-parametric bootstrapping with 5,000 samples; unstandardised estimates are identical whether the mediator residual covariance is estimated or fixed to zero. All effects significant at *p* < 0.001. The contrast row reports a formal test of the difference between the two indirect effects.

Both a-paths were statistically significant (*p* < 0.001), indicating that higher Understanding was associated with greater Confidence (β = 0.461) and greater Competence (β = 0.426). Understanding was associated with both mediators with comparable strength, suggesting it functions as a broad cognitive foundation related to both affective and skill-related self-perceptions. Both b-paths were also significant (*p* < 0.001), with Confidence (β = 0.467) showing a notably stronger association with Enjoyment than Competence (β = 0.217).

Both indirect effects were positive and statistically significant, with bootstrapped confidence intervals excluding zero. The indirect effect through Confidence (Unstd. = 0.283, Std. β = 0.216, 95% CI [0.206, 0.369]) was more than double that through Competence (Unstd. = 0.122, Std. β = 0.093, 95% CI [0.073, 0.173]). A formal bootstrap contrast confirmed that the confidence-mediated indirect effect was significantly larger than the competence-mediated indirect effect (difference in unstandardised indirect effects = 0.161, SE = 0.056, *z* = 2.87, *p* =.004, 95% CI [0.053, 0.272]; standardized difference = 0.123), with the interval excluding zero, supporting the interpretation of Confidence as the stronger mediating pathway across the full sample. When the model was re-estimated with cluster-robust standard errors accounting for the nesting of pupils within schools (section 2.6), this contrast was not statistically significant (*p* = 0.149), although both indirect effects individually retained significance. The claim that Confidence is the stronger mediator is therefore supported under conventional estimation but is not robust to the most conservative treatment of the clustered sampling design, and is reported with that qualification. This difference reflects the relative strength of the b-paths rather than the a-paths: understanding was associated with both mediators similarly, but Confidence was considerably more strongly related to Enjoyment.

The direct effect of Understanding on Enjoyment remained significant after accounting for both mediators (Unstd. = 0.326, Std. β = 0.248, 95% CI [0.240, 0.411]), indicating partial rather than full mediation. The total indirect effect (Std. β = 0.308) exceeded the direct effect (Std. β = 0.248), suggesting that the majority of the association between Understanding and Enjoyment operates through the mediating roles of Confidence and Competence, with Confidence accounting for the larger share.

### 3.5 Model fit

The baseline parallel path model returned poor global fit indices (χ^2^ (1) = 252.85, *p* < 0.001, CFI = 0.778, RMSEA = 0.629, SRMR = 0.166). This reflects a single constraint: the model fixes the residual covariance between Confidence and Competence to zero, yet the two are correlated at *r* =.66 and Understanding does not account for all of their shared variance. Freeing this covariance yields a just-identified model that reproduces the data exactly, and because each structural equation is already a saturated regression, the unstandardised path estimates are unchanged (identical to eight decimal places); only the standardized coefficients differ slightly, as standardization depends on the model-implied variance of Enjoyment. The standardized estimates in Table 6 are from this model, in which the residual correlation between the mediators was substantial (0.572, *p* < 0.001). Because single-item indicators preclude separation of measurement from structural components (section 2.3), interpretation throughout rests on the structural path coefficients, indirect effects, and likelihood ratio tests rather than on global fit indices.

### 3.6 Moderated mediation models

Moderated mediation analyses were conducted using multi-group structural equation modeling to examine whether the mediation patterns varied across key demographic and contextual groups. As noted above, these analyses were exploratory. Separate models were estimated for gender, ethnicity, PAQ-C activity level, school setting, and SEN status. Overall moderation was evaluated using likelihood ratio tests comparing constrained and unconstrained models, with group-specific indirect effects estimated via Monte Carlo simulation (10,000 draws). Results are presented in Table 7, and group-specific indirect effects for the significant moderators are displayed in Figure 2.

**Figure 2:**
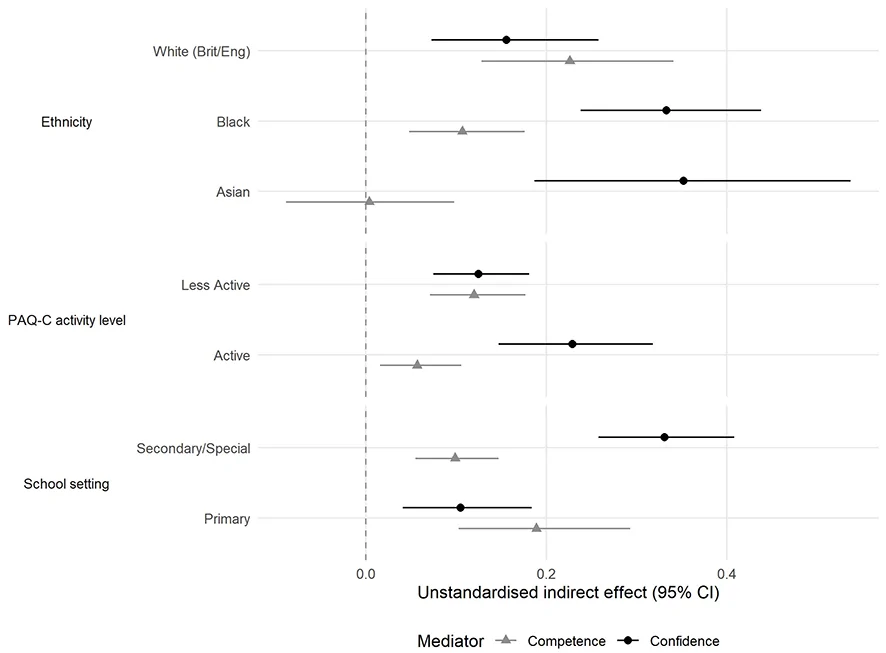
Group-specific indirect effects of Understanding on Enjoyment via Confidence and Competence, for the three significant moderators (*N* = 637). Points are unstandardised indirect effects with 95% confidence intervals derived from Monte Carlo simulation (10,000 draws) based on group-specific path estimates from the multi-group models. Black circles denote the Confidence pathway and gray triangles the Competence pathway. The dashed vertical line marks zero; an indirect effect is statistically significant where its interval excludes zero. Secondary/Special combines mainstream secondary (*n* = 406) and special school (*n* = 82) pupils. Gender and SEN status are not shown, as neither moderated the mediation pathways. Subgroup moderation analyses were exploratory. Figure generated from lavaan model output.

**Table 7:** Moderated mediation results for the understanding model.

A. Likelihood ratio tests for overall moderation of regression paths									
Moderator		χ^2^diff		*df*	*p*-value		Conclusion		
Gender		5.58		5	0.349		No moderation		
SEN		0.43		5	0.994		No moderation		
PAQ-C activity level		21.76		5	< 0.001		Significant moderation		
School setting		19.79		5	0.001		Significant moderation		
Ethnicity		25.48		10	0.005		Significant moderation		
B. Group-specific indirect effects for significant moderators									
Moderator	Group	Indirect via confidence (Unstd.)	95% CI	Std. β	Indirect via competence (Unstd.)	95% CI	Std. β	Contrast (Std.)	*P*
PAQ-C	Less active	0.125	[0.055, 0.214]	0.104	0.120	[0.057, 0.195]	0.101	0.003	0.955
PAQ-C	Active	0.229	[0.145, 0.327]	0.184	0.057	[0.010, 0.117]	0.045	0.140	0.001
School setting	Primary	0.104	[0.017, 0.231]	0.092	0.190	[0.079, 0.329]	0.163	−0.071	0.302
School setting	Secondary/special	0.331	[0.255, 0.416]	0.242	0.099	[0.049, 0.155]	0.072	0.170	< 0.001
Ethnicity	White (British/English)	0.156	[0.050, 0.301]	0.109	0.226	[0.119, 0.360]	0.155	−0.046	0.583
Ethnicity	Asian/Asian British	0.352	[0.159, 0.588]	0.259	0.005	[−0.112, 0.122]	0.004	0.255	0.006
Ethnicity	Black/Black British	0.334	[0.220, 0.469]	0.264	0.106	[0.037, 0.186]	0.078	0.186	0.012

Unstandardised indirect effects and 95% CIs derived from Monte Carlo simulation (10,000 draws) based on group-specific path estimates and standard errors from the 5,000 bootstrap multi-group models. Standardized point estimates (Std. β) are from the multi-group models in which the residual covariance between the mediators is freely estimated (section 3.5). The Contrast column reports the standardized difference between the two indirect effects within each group, with its associated p-value; a non-significant contrast indicates that the two pathways do not differ reliably in that group. A significant indirect effect is indicated where the 95% CI excludes zero. Gender and SEN are omitted from Panel B as neither moderated any pathway significantly. Secondary or Special combines secondary school (*n* = 406) and special school (*n* = 82) pupils. Subgroup moderation analyses were exploratory.

### 3.7 Moderation by gender

Gender did not significantly moderate the regression paths in either mediation model (χ^2^ diff(5) = 5.58, *p* = 0.349). Confidence- and competence-mediated effects were statistically equivalent for boys and girls, and all univariate score tests were non-significant (all *p* > 0.20). Despite small-to-moderate descriptive differences in mean levels of Enjoyment, Confidence, and Competence favoring boys, the underlying patterns linking Understanding to Enjoyment operated similarly across genders. These findings suggest that while boys and girls may differ in their average levels of physical activity-related perceptions, the cognitive-affective associations through which Understanding relates to Enjoyment are broadly invariant across gender.

### 3.8 Moderation by SEN status

SEN status did not significantly moderate any regression paths (χ^2^ diff(5) = 0.43, *p* = 0.994), and all univariate score tests were non-significant (all *p* > 0.74). The group-specific indirect effects were virtually identical across SEN and No SEN groups for both the Confidence pathway (No SEN: Unstd. = 0.273, Std. β = 0.210; SEN: Unstd. = 0.290, Std. β = 0.222) and the Competence pathway (No SEN: Unstd. = 0.116, Std. β = 0.088; SEN: Unstd. = 0.134, Std. β = 0.103). These findings indicate that the cognitive-affective associations linking Understanding to Enjoyment did not differ detectably by SEN status as recorded here. This is a reassuring pattern given the descriptive differences observed between these groups, though it should be read in light of the composition of the SEN group, the majority of whom attended the specialist setting described in section 2.2 (see Limitations).

### 3.9 Moderation by PAQ-C activity level

PAQ-C activity level significantly moderated the overall pattern of regression paths (χ^2^ diff) = 21.76, *p* < 0.001). Univariate score tests identified two specific paths as driving this moderation: the Understanding → Competence a-path (χ^2^ (1) = 5.62, *p* = 0.018) and the Confidence → Enjoyment b-path (χ^2^(1) = 6.66, p = 0.010). The difference between activity groups therefore involves both the competence a-path and the confidence b-path rather than a single pathway, consistent with the group-specific indirect effects reported below, in which the Confidence pathway strengthens and the Competence pathway weakens among Active pupils.

Group-specific indirect effects revealed a meaningful difference in the pattern of mediation. For Less Active pupils, the Confidence and Competence pathways contributed comparably to the indirect association of Understanding with Enjoyment (Confidence: Unstd. = 0.125, Std. β = 0.104, 95% CI [0.055, 0.214]; Competence: Unstd. = 0.120, Std. β = 0.101, 95% CI [0.057, 0.195]), and the standardized contrast between them was negligible and non-significant (0.003, *p* = 0.955). For Active pupils, however, the Confidence pathway was considerably stronger (Unstd. = 0.229, Std. β = 0.184, 95% CI [0.145, 0.327]), while the Competence pathway was notably weaker and only marginally significant (Unstd. = 0.057, Std. β = 0.045, 95% CI [0.010, 0.117]), a difference that was statistically reliable (standardized contrast = 0.140, *p* = 0.001). This pattern is consistent with the possibility that physical activity experience is associated with a greater relative role for Confidence as a mediating mechanism and a reduced relative contribution of perceived Competence, though the cross-sectional design precludes firm conclusions about direction.

It is worth noting that Active pupils and Primary pupils overlap substantially: primary pupils had a mean PAQ-C score of 3.61 compared with 2.75 for Secondary/Special pupils (*d* = 1.06). This overlap is evident at the level of individual schools (Table 1): 85.1 and 85.7% of pupils in the two primary schools were classified as Active, compared with 46.8, 44.1 and 40.0% in the two secondary schools and the special school respectively. The distinction between activity groups therefore aligns closely with the distinction between educational phases, and is consistent within phase rather than varying between schools of the same type. Consequently, the patterns observed for activity level and school setting are not fully independent and should be interpreted with appropriate caution. The moderation analyses for each factor were conducted separately and cannot establish the relative contributions of activity level and developmental stage as independent moderators. Future research using designs that disentangle these factors is needed.

### 3.10 Moderation by school setting

School setting significantly moderated the overall pattern of regression paths (χ^2^ diff(5) = 19.79, *p* = 0.001). Univariate score tests identified two specific paths as driving this moderation: the Understanding → Confidence a-path (χ^2^ (1) = 7.63, *p* = 0.006) and the Confidence → Enjoyment b-path (χ^2^(1) = 6.97, *p* = 0.008). Both significant paths therefore involve Confidence, indicating that the difference between settings is concentrated in the confidence pathway; the Understanding → Competence a-path did not reach significance (χ^2^(1) = 3.52, p = 0.061).

Group-specific indirect effects revealed a notable shift in the relative contribution of the two mediating pathways across school settings. In Primary settings, the Competence pathway was the stronger mediator (Unstd. = 0.190, Std. β = 0.163, 95% CI [0.079, 0.329]), while the Confidence pathway was weaker and its CI only narrowly excluded zero (Unstd. = 0.104, Std. β = 0.092, 95% CI [0.017, 0.231]). In Secondary or Special settings, the Confidence pathway was substantially stronger (Unstd. = 0.331, Std. β = 0.242, 95% CI [0.255, 0.416]), while the Competence pathway was more modest (Unstd. = 0.099, Std. β = 0.072, 95% CI [0.049, 0.155]). This is consistent with a shift in the relative importance of the two mediators across school settings. However, as noted in section 3.9, Primary pupils also reported substantially higher PAQ-C scores, and the concurrent moderation by activity level means that this school-setting pattern cannot be attributed to developmental stage alone; it is interpreted as a tentative, exploratory finding (see section 4.3).

### 3.11 Moderation by ethnicity

Ethnicity significantly moderated the overall pattern of regression paths (χ^2^ diff(10) = 25.48, *p* = 0.005), but no single path reached significance in the univariate score tests (all *p* > 0.12), and pairwise differences between ethnic groups in specific indirect effects were not significant. The group-specific patterns are therefore offered as exploratory observations rather than confirmed pathway-level differences. Full group-specific indirect effects are reported in Table 7. In brief, the competence pathway appeared the stronger mediator for White British or English pupils, whereas the confidence pathway dominated for Asian or British Asian pupils (with the competence pathway close to zero) and for Black or Black British pupils.

A more serious interpretive constraint arises from the distribution of ethnic groups across schools. Ethnicity and school are substantially confounded: 93.3% of Black or Black British pupils attended a single secondary school (School C), so this group is in practical terms a single-school sample; Asian or British Asian pupils were more evenly spread across three schools and two phases (no more than 35.4% in any one), and White British or English pupils across three schools (maximum 41.2%), although 33.5% attended the special school (School E), which was 90.2% White British. The extent to which each group-specific result reflects ethnicity rather than school therefore varies markedly across groups. To address this directly, a sensitivity analysis estimating the ethnicity model within a single school is reported in section 3.12.

### 3.12 Sensitivity analyses

Four sensitivity analyses tested the robustness of the setting, SEN, and ethnicity results. First, excluding special school pupils and comparing Primary (*n* = 149) with mainstream Secondary pupils only (*n* = 406) left the setting moderation significant and the pattern unchanged (χ^2^ diff(5) = 20.94, *p* < 0.001), with competence the stronger mediator in Primary settings and confidence dominant in mainstream Secondary settings; the setting difference in section 3.10 is therefore not an artifact of including the special school. Second, a three-group model (Primary, Secondary, Special) was also significant (χ^2^ diff(10) = 26.43, *p* =.003), with School E resembling the primary rather than the mainstream secondary pattern; a direct Secondary-vs.-Special comparison, in which both groups occupy the same educational phase and age range so that any difference between them is not confounded with developmental stage, was not significant (χ^2^ diff(5) = 6.36, *p* = 0.273), which, given the special school subgroup size (*n* = 82), indicates an absence of detectable difference rather than equivalence and supports pooling these groups in the main analysis. Third, recoding all special school pupils as having SEN on the basis of their EHCP-based placement (section 2.2) produced substantively identical results (χ^2^ diff(5) = 0.66, *p* = 0.985), so the null moderation by SEN status does not depend on the self-report coding of that variable.

Fourth, to address the confounding of ethnicity with school, the ethnicity model was re-estimated within School C alone, which contained all three analytic groups in usable numbers (White British *n* = 91, Asian *n* = 60, Black *n* = 98) and holds setting, catchment, curriculum and staffing constant. Ethnicity did not moderate the pathways within School C (χ^2^ diff(10) = 8.53, *p* = 0.578; all univariate score tests *p* > 0.08), and the group-specific profiles were markedly more similar than in the full sample; in particular, the competence-dominant pattern observed for White British pupils in the full sample was absent, their confidence pathway exceeding their competence pathway as it did for Asian and Black pupils in the same school. This analysis is underpowered (*n* = 60–98 per group) and cannot establish the absence of an ethnicity effect, but it provides no evidence of one independent of school, consistent with a compositional account of the full-sample moderation (section 4.5).

### 3.13 Intersectional considerations

The significant moderators overlap substantially: the confidence-dominant pattern in Secondary or Special settings coincides with the stronger confidence pathways seen for Active pupils and for Asian and Black pupils, and school setting, activity level, and ethnic composition are themselves interrelated. The moderation effects are therefore not independent, and some between-group differences may reflect shared rather than unique variance. Sample sizes did not permit full intersectional modeling, so these observations remain speculative; disentangling how demographic and contextual factors jointly shape these mechanisms would require larger, more deliberately sampled subgroups and multilevel or intersectional designs.

## 4 Discussion

The present study examined the cognitive and affective correlates of pupils' enjoyment of physical activity, and the extent to which these patterns varied across demographic and contextual groups. Drawing on Self-Determination Theory (11) and physical literacy frameworks (19), the findings provide new insights into how understanding, confidence, and competence relate to young people's experiences of physical activity. The results highlight both broadly consistent patterns and exploratory subgroup differences, offering a nuanced picture of the factors associated with enjoyment, and, through enjoyment, with habitual physical activity participation.

### 4.1 Understanding, confidence, and enjoyment

Across the full sample, the path analysis model demonstrated that pupils' cognitive understanding of physical activity was indirectly associated with enjoyment through both confidence and competence. Pupils who better understood why physical activity was beneficial tended to feel more confident and competent, and these perceptions were in turn associated with greater enjoyment. This pattern aligns with SDT, which posits that perceived competence is a fundamental psychological need supporting intrinsic motivation and positive affective experiences (11), and with physical literacy frameworks that identify understanding as a foundational cognitive component of lifelong PA engagement (17, 19).

Confidence emerged as the stronger mediator in both the baseline model and across most moderation analyses. The indirect effect through confidence (Std. β = 0.216) was more than double that through competence (Std. β = 0.093), and a formal bootstrap contrast confirmed that this difference was statistically reliable (standardized difference = 0.123, *p* < 0.001), suggesting that pupils' belief in their ability to participate in physical activity is more strongly associated with enjoyment than their perceived skill level across the full sample. This distinction echoes findings from physical literacy research emphasizing confidence as a core driver of motivation and sustained participation (15). The direct association of understanding with enjoyment also remained significant after accounting for both mediators, indicating partial rather than full mediation and suggesting that understanding is also related to enjoyment beyond its associations with confidence and competence.

### 4.2 Enjoyment and physical activity level

Enjoyment alone accounted for 28.4% of the variance in PAQ-C scores, and in a multiple regression including all four psychological constructs, enjoyment and confidence emerged as equally strong independent correlates of activity level (both β ≈ 0.27), while understanding was not a significant independent correlate once affective variables were accounted for. This pattern suggests that enjoyment and confidence are the most proximal psychological correlates of habitual physical activity, while understanding appears to operate more distally, relating to activity levels indirectly through its associations with affective mechanisms rather than predicting behavior independently.

These findings are consistent with theoretical arguments that enjoyment functions as a gateway to sustained PA participation (6) and extend them by showing that confidence is associated with activity behavior as strongly as enjoyment. Together, these results suggest that interventions targeting both enjoyment and confidence, rather than focusing narrowly on increasing activity volume, may be more effective in supporting long-term PA engagement. However, the cross-sectional design means the direction of these associations cannot be established. Pupils who are more active may report higher enjoyment and confidence as a consequence of their participation, rather than enjoyment and confidence preceding behavior change. Longitudinal research is needed to establish these relationships.

### 4.3 Moderation by school setting

The clearest exploratory moderation finding concerned school setting, where the relative contribution of the two mediating pathways differed by phase. In Primary settings, the competence pathway was numerically the stronger mediator (Std. β = 0.163) and the confidence pathway weaker (Std. β = 0.092), although the two did not differ significantly (standardized contrast = −0.071, *p* = 0.302). In Secondary or Special settings, confidence was the dominant pathway (Std. β = 0.242), significantly exceeding competence (Std. β = 0.072; standardized contrast = 0.170, *p* < 0.001). This finding is noteworthy given that Primary pupils reported higher mean scores across all variables, suggesting that the way understanding relates to enjoyment may shift in nature, not just in magnitude, across school settings.

One interpretation of this pattern is developmental. For primary-aged children, enjoyment may be more closely tied to whether activity feels manageable and within reach, the sense of ease that the competence item captures, reflecting a stage in which finding activity approachable is central to positive PA experiences (39). As pupils enter secondary education, the social landscape of physical activity becomes more complex: peer comparison, self-consciousness, and identity-based appraisals of PA become increasingly salient during adolescence (39), which could shift the psychological basis of enjoyment away from perceived competence and toward broader self-belief and confidence. On this reading, secondary pupils rely more heavily on confidence to navigate the more demanding social and physical environments they encounter.

This developmental account is plausible and consistent with the observed pattern, but it is one possibility among several and cannot be established from the present data. School setting is confounded with physical activity level, with Primary pupils reporting PAQ-C scores approximately one standard deviation higher than Secondary/Special pupils (*d* = 1.06); it is also confounded with individual school, since the two settings comprise different schools with different intakes, cultures, and provision, and the Secondary/Special group is dominated by a single school. A developmental shift, a difference in habitual activity level, and the particular characteristics of the specific schools sampled are not separable here, and any of them could underlie the result. The finding is therefore best read as a contextual difference in how understanding relates to enjoyment across these settings, whose developmental or non-developmental basis remains open. Distinguishing these explanations would require designs that separate developmental stage from school context and activity level, such as longitudinal tracking within schools or activity-matched samples across phases.

Notwithstanding this caution, the practical reading is reasonably stable: in primary settings, pedagogical approaches that prioritize skill development, mastery experiences, and task-oriented feedback may best support enjoyment, whereas in secondary settings the emphasis may need to shift toward building broader self-belief and confidence through autonomy-supportive teaching, positive social climates, and opportunities for personal challenge.

### 4.4 Moderation by PAQ-C activity level

Activity level also moderated the mediation patterns, complementing the school setting findings. For Active pupils, the confidence pathway was significantly stronger than the competence pathway (Std. β = 0.184 vs. 0.045; standardized contrast = 0.140, *p* = 0.001). For Less Active pupils, the two pathways contributed almost identically and did not differ (confidence Std. β = 0.104, competence Std. β = 0.101; standardized contrast = 0.003, *p* = 0.955). This is consistent with the possibility that greater physical activity experience is associated specifically with a stronger role for confidence, and a reduced relative contribution of perceived competence.

One interpretation is that Active pupils have accumulated sufficient physical activity experience that their engagement is less contingent on feeling skilled and more strongly anchored in a broader sense of confidence and self-efficacy. For Less Active pupils, perceived competence remains an equally important correlate through which understanding relates to enjoyment, suggesting that helping them feel more at ease with activity may be particularly important for engaging this group. As above, the cross-sectional design means the direction of these associations cannot be established.

### 4.5 Moderation by ethnicity

Ethnicity moderated the overall pattern of regression paths, but no single path reached significance in the univariate score tests, so the group-specific effects are interpreted only as exploratory. In the full sample, competence appeared the stronger mediator for White British or English pupils (though the within-group contrast was not significant, *p* = 0.583), while confidence dominated for Asian or British Asian pupils (contrast *p* = 0.006) and Black or Black British pupils (contrast *p* = 0.012), with the competence pathway close to zero for Asian pupils.

These apparent differences are substantially confounded with school. Ethnic groups were unevenly distributed across the five schools: 93.3% of Black or Black British pupils attended a single secondary school, and 57.9% of White British pupils attended either the special school or one of the two primary schools, settings in which the competence pathway plays a larger role than in mainstream secondary provision (section 3.12). A sensitivity analysis estimating the ethnicity model within a single school, which holds setting, catchment, curriculum and staffing constant, indicated that ethnicity did not moderate the pathways once school was held constant (χ^2^ diff(10) = 8.53, *p* = 0.578); notably, the competence-dominant pattern for White British pupils disappeared, their profile closely resembling that of Asian and Black pupils in the same school. Although underpowered (*n* = 60–98 per group), this analysis provides no evidence of an ethnicity effect independent of school. The most parsimonious account of the full-sample moderation is therefore compositional, reflecting where pupils from each group were educated rather than ethnicity itself, and the group differences in section 3.11 should not be read as demonstrating culturally patterned mechanisms. Interpretations sometimes advanced for such patterns, such as self-presentation or modesty norms attenuating reported competence among some South Asian pupils (40), are not supported here and would require a design that separates ethnicity from school context.

One broader contextual point nonetheless deserves emphasis. All pupils experienced physical education framed by the National Curriculum, whose content and norms scholars have argued are oriented toward White British pupils, with documented limits for engaging pupils from ethnically diverse backgrounds (23, 41). Where the activities, success criteria, and bodily ideals that define “being good at” PE reflect a culturally specific norm, perceived competence may carry different meanings, and bear a different relationship to enjoyment, for pupils whose movement cultures differ from that norm. This is relevant to interpreting competence and enjoyment even though the present data cannot isolate ethnicity from school, and it reinforces the case for culturally responsive provision discussed below. Consistent with the importance of not aggregating across minority groups, the descriptive data showed that Asian pupils scored higher than White British pupils across all four constructs whereas Black and White pupils did not differ, suggesting that different minority groups may reach comparable outcomes by different routes.

### 4.6 Moderation by gender and SEN

Gender did not moderate any mediation pathways, despite small-to-moderate descriptive differences favoring boys on Enjoyment, Confidence, and Competence. This suggests that while boys and girls differ in their mean levels of PA-related perceptions, the psychological associations through which understanding relates to enjoyment operate similarly across genders, consistent with prior work showing broadly comparable motivational processes in PE contexts (39, 41). This finding contributes to a growing body of evidence challenging simplistic gender narratives in physical activity research and underscores the importance of moving beyond mean-level comparisons toward more nuanced analyses of the mechanisms underlying PA engagement.

SEN status also did not moderate any mediation pathways, and the group-specific indirect effects were virtually identical across SEN and No SEN groups. This is an encouraging pattern: despite meaningful descriptive differences showing that pupils with SEN reported significantly lower scores on all four variables, the cognitive-affective associations linking understanding to enjoyment appear to operate similarly across these groups. This tentatively suggests that interventions targeting these pathways, for example through building understanding and confidence, may be broadly applicable to pupils with and without SEN, though the lower baseline levels observed for pupils with SEN indicate that additional support may be needed to help this group access and benefit from such approaches. Two caveats temper this interpretation. First, special school pupils were combined with secondary pupils for analysis, which may obscure differences between mainstream and special school contexts. Second, and more consequentially, the majority of pupils recorded as having SEN attended a single special school, so the SEN contrast is partly a contrast between that specific setting and the remainder of the sample, and the SEN category itself spans a heterogeneous range of needs and provision (see Limitations). The null result is therefore better understood as an absence of detectable difference between coarsely defined groups than as evidence of equivalent mechanisms across the full diversity of special educational needs.

### 4.7 Implications for practice

The findings carry several practical implications for schools, practitioners, and policymakers seeking to enhance pupils' enjoyment of and engagement with physical activity. Because they derive from cross-sectional associations, these implications are best read as theoretically informed directions for practice and intervention design rather than as established intervention effects.

Confidence-building should be a central priority in PA provision, particularly in secondary settings, where it consistently emerged as the dominant mediating mechanism. Strategies such as structured success experiences, positive and process-focused feedback, and opportunities for personal challenge and mastery are likely to be valuable. However, given the central role of social comparison and self-consciousness in adolescence, we encourage practitioners and policymakers to be more ambitious than a default reliance on established mastery (task-involving) motivational climates. While mastery climates remain a sound foundation, motivational-climate interventions have been advocated for decades without consistently shifting population-level engagement, and the secondary-school confidence finding suggests the more pressing practical problem is reducing the *salience of social comparison itself* . Several concrete directions are worth exploring: (i) moving from mastery climates toward explicitly empowering climates (in the sense of autonomy-supportive, task-involving, and relatedness-supportive provision combined), which more deliberately protect pupils' sense of agency and belonging; (ii) curricular and assessment choices that de-emphasize public ranking, visible ability grouping, and whole-class performance display, which structurally generate comparison; (iii) broadening the activity offer beyond traditional competitive team sports toward lifestyle, adventurous, aesthetic, and self-referenced activities in which “success” is less zero-sum; (iv) giving pupils genuine choice and voice over activities and how progress is judged, shifting the reference point from peers to personal progress; and (v) staff development focused specifically on the language of feedback and grouping practices that inadvertently heighten comparison. These are proposed as testable directions rather than proven solutions, and we suggest they are evaluated directly in future intervention research.

In primary settings, a complementary emphasis on skill development, task mastery, and perceived competence is indicated. The difference in mediating pathways across settings suggests that context-tailored approaches, emphasizing competence-building in primary settings and confidence-building in secondary settings, may be more effective than uniform strategies; this is offered as a practical direction consistent with the observed pattern rather than as evidence of a developmental progression, given that setting is confounded with activity level and with individual school.

Developing pupils' understanding of physical activity represents an accessible and often overlooked entry point for improving enjoyment and engagement. Integrating brief educational components into PA provision, explaining why activities are beneficial, connecting movement to health and wellbeing outcomes, or helping pupils understand what they are developing through participation, may strengthen the cognitive foundations associated with confidence and ultimately enjoyment. The findings also suggest that understanding does not need to translate directly into behavior change to be valuable; its primary contribution may be in nurturing the affective processes that make physical activity a positive experience.

For less active pupils, both confidence and competence contribute comparably to the understanding–enjoyment association, suggesting that helping pupils feel both capable and at ease with activity, alongside building confidence, may be especially important for engaging those who are currently least active. Targeted approaches that provide positive, low-pressure introductions to a range of physical activities, emphasizing inclusion, choice, and personal progress, may help Less Active pupils develop the affective foundations that support sustained engagement.

The ethnic differences observed in specific mediation pathways, though exploratory, highlight the importance of culturally responsive practice that reflects the diverse backgrounds of pupils. The findings are consistent with the possibility that the same outcomes, confidence, competence, and enjoyment, may be reached through different psychological routes for pupils from different ethnic backgrounds, reinforcing the need for flexible and adaptive rather than one-size-fits-all approaches. Given the culturally specific orientation of the National Curriculum noted above, practitioners should be supported to recognize and respond to the varied cultural contexts, movement cultures, expectations, and prior experiences pupils bring to PA, and to broaden definitions of valued activity and success accordingly.

The descriptive data for pupils identifying as gender-diverse (“Other”) showed notably lower scores across all constructs despite habitual activity levels comparable to the sample as a whole, though sample sizes precluded formal analysis. These patterns represent a potential equity concern, compounded by the fact that the gender-specific PAQ-C thresholds structurally excluded these pupils from the activity-level analyses. Practitioners should consider how PA environments and social climates may affect the experiences of gender-diverse young people; systematic review evidence indicates that trans and non-binary pupils frequently report negative experiences in physical education, particularly where provision is organized around binary gender categories (42). Future research should also adopt activity measures and classification procedures that do not exclude pupils on the basis of a binary gender split.

Finally, the robust association between enjoyment and habitual physical activity levels reinforces the case for centring enjoyment as a primary outcome in its own right, not merely as a means to an end, but as a valuable indicator of positive PA experiences that is likely to sustain engagement over time. Schools and policymakers should resist the temptation to prioritize activity-volume metrics at the expense of the affective quality of pupils' PA experiences and should invest in provision that makes physical activity genuinely enjoyable for all pupils regardless of background, ability, or current activity level.

### 4.8 Limitations

Several limitations should be acknowledged when interpreting the findings. Although the mediation models are theoretically grounded, the direction of effects cannot be established from the present cross-sectional data. In particular, the associations between enjoyment, confidence, and physical activity level are consistent with the theoretical model but could equally reflect reverse causation: pupils who are more active may develop higher enjoyment and confidence as a consequence of their participation rather than these factors preceding behavior change. Longitudinal and experimental designs are needed to establish the causal structure of these relationships.

The study relied on single-item measures for all four psychological constructs. As detailed in section 2.3, this prevents measurement error from being modeled separately from structural paths, meaning the analyses are best characterized as path analysis rather than latent variable SEM. In addition, two specific items have interpretive limits: the competence item (“I find exercise and sports easy”) captures perceived ease, which overlaps with but is distinct from perceived skill or capability, and the understanding item showed a ceiling-leaning distribution that may attenuate associations involving understanding. Future research should replicate these findings using validated multi-item scales to provide more robust measurement of the constructs; recent reviews of physical literacy assessments designed for school-aged children provide guidance on instruments that combine adequate validity with feasibility in school settings (43).

Pupils were nested within only five schools of markedly uneven size, one contributing more than half the sample. Intraclass correlations were modest (0.014–0.055), and design-effect analysis indicated that clustering had little effect on the mediation paths and indirect effects that carry the study's main conclusions, which were estimated almost entirely from within-school variation (effective sample sizes of approximately 420 to 580), but a larger effect on the precision of the descriptive and subgroup mean comparisons (effective sample sizes of approximately 50–156), which are interpreted with corresponding caution. Under cluster-robust estimation all individual paths and both indirect effects remained significant; the single exception was the contrast between the two indirect effects (section 3.4). With so few clusters this correction is itself imprecise (44), and is best read as indicating where a conclusion is sensitive to the treatment of clustering rather than as a definitive adjustment. The conclusion that confidence is the stronger mediator would accordingly benefit from replication in a design with a larger number of schools. The small number of schools, and the concentration of the sample within them, also limit the national representativeness of the findings.

This clustering has a direct consequence for the school-setting moderation: with two primary schools on one side and three secondary or special schools on the other, the contrast between settings is not separable from the contrast between individual schools, and could equally reflect their particular intakes, staffing, or provision. The finding is therefore best read as a difference between two groups of schools that differ in phase, not as a demonstrated effect of educational phase itself (section 4.3); establishing the latter would require sampling many schools within each setting.

Several features of the SEN comparison warrant caution. SEN status was based on pupil self-report rather than administrative records: in the special school, where all pupils are placed following an Education, Health and Care Plan, only 86.6% self-identified as having SEN, and in mainstream settings 10.3% self-identified against national figures of roughly 21% for pupils with identified needs. The No SEN comparison group therefore likely contains pupils with unrecognized needs, attenuating the SEN contrast and biasing it toward the null, so the non-significant moderation by SEN status is weaker evidence of equivalence than it appears. The SEN group is also confounded with a single setting: 71 of the 128 pupils identifying as having SEN attended the special school (School E), which is atypical on several dimensions simultaneously (90.2% White British, 15.9% girls, and the lowest activity levels in the sample; Table 1). SEN status is thus partly a contrast between that specific school and the remainder of the sample, and the binary SEN flag groups pupils in specialist provision with mainstream pupils whose needs and support differ substantially. The null moderation for SEN should accordingly be read as an absence of detectable difference between coarsely defined, imperfectly measured groups rather than as evidence of equivalent mechanisms across the full range of special educational needs. Replication using administratively verified SEN status, across special schools with differing curricular emphases, would help establish how far these findings generalize.

Several key moderators are substantially confounded in this sample. School setting and physical activity level overlap considerably (Primary vs. Secondary/Special: *d* = 1.06 on PAQ-C), meaning that findings attributed to school setting may partly reflect activity-level differences, and vice versa. Similarly, gender and activity level are partially confounded (boys reported higher PAQ-C scores: *d* = 0.48), and ethnic group composition may co-vary with school setting. These confounds limit the interpretability of individual moderation effects and should be addressed in future research through designs that allow simultaneous estimation of competing moderators, or through sampling strategies that deliberately vary these factors independently.

Ethnicity was likewise confounded with school: because ethnic groups were unevenly distributed across settings, the ethnicity moderation cannot be separated from school composition, and a within-school sensitivity analysis found no ethnic-group differences once school was held constant (sections 3.12 and 4.5). Only the three largest ethnic groups could be modeled, leaving Mixed-Race pupils and others unexamined, and the small numbers of gender-diverse pupils prevented formal analysis of that group. The subgroup analyses more generally are likely underpowered for smaller groups and for detecting pathway-specific differences, which is the basis for treating all subgroup results as exploratory.

Finally, physical activity was assessed using the PAQ-C, a self-report measure that may be influenced by recall bias or social desirability, references only the previous seven days, and does not provide objective data on activity type, intensity, or duration. The use of a children's instrument toward the upper end of the 8–16 age range is a further limitation. Future studies should consider supplementing self-report PA measures with accelerometry or other objective measures to provide a more accurate picture of pupils' habitual activity levels.

## 5 Conclusion

This study contributes to a growing body of work emphasizing the central role of enjoyment in shaping young people's engagement with physical activity. Drawing on a large and diverse sample of school-aged pupils, the findings are consistent with a model in which understanding functions as a distal cognitive correlate associated with confidence and competence, which are in turn associated with enjoyment and with habitual physical activity participation. Critically, the finding that understanding does not independently predict activity level, but is related to it indirectly through confidence and competence, helps clarify the theoretical position of cognitive factors in the PA motivation chain and supports a model consistent with both self-determination theory and physical literacy frameworks, in which affective factors are the more proximal correlates of behavior and cognitive foundations play a more distal but nonetheless meaningful role.

The study also provides important nuance by demonstrating that these patterns, while broadly consistent across gender and SEN status, varied in their relative strength according to school setting, physical activity level, and ethnicity. The most novel exploratory contribution concerns the dominant mediating pathway across school settings: confidence is the dominant pathway in secondary and special school settings, significantly exceeding competence, whereas in primary settings the two pathways contribute comparably and do not differ reliably. This suggests that the psychological basis of enjoyment may differ across school contexts, a pattern that, to our knowledge, has not previously been documented in the physical literacy or SDT literature. Whether this reflects developmental stage, accumulated activity experience, or the characteristics of the specific settings sampled cannot be determined from the present cross-sectional, school-confounded data, and the pattern merits investigation in designs able to separate these influences. The exploratory ethnic differences observed in specific pathways similarly highlight the need for culturally responsive pedagogies that acknowledge the varied experiences and expectations pupils bring to physical activity.

The absence of moderation by gender and SEN status, despite meaningful descriptive differences between these groups, suggests that the underlying psychological associations linking understanding to enjoyment operate similarly across them, adding to a growing body of evidence challenging simplistic categorical narratives in physical activity research. The consistently lower scores observed for pupils with SEN across all variables, and the descriptive patterns for gender-diverse pupils, underscore the continued need for targeted support and dedicated research to ensure these groups can access the cognitive and affective foundations that support enjoyment and participation.

These findings highlight the value of moving beyond narrow, volume-based conceptions of physical activity toward a more holistic understanding of what is associated with young people's engagement. Enjoyment, related to understanding, confidence, and competence, emerges as both a powerful correlate of immediate engagement and a likely contributor to longer-term physical activity habits. By foregrounding these affective and cognitive dimensions, and by adapting provision to the setting, activity experience, and cultural context of different pupil groups, educators and policymakers can better support pupils in developing positive, meaningful, and sustainable relationships with physical activity.

## Data Availability

The datasets presented in this article are not readily available because access to this dataset is restricted due to its sensitive nature and because it is derived from a larger dataset generated as part of a research project funded by a UK Charity. In accordance with the terms and conditions of that funding and the ethical obligations associated with the original study, the data cannot be made publicly available. Requests for access may be directed to the corresponding author, who will assess eligibility in line with the applicable data governance framework. Requests to access the datasets should be directed to t.vernon@shu.ac.uk.
